# Repetitive finger movement performance differs among Parkinson’s disease, Progressive Supranuclear Palsy, and spinocerebellar ataxia

**DOI:** 10.1186/s40734-014-0015-y

**Published:** 2015-02-16

**Authors:** Elizabeth L Stegemöller, Jennifer Uzochukwu, Mark D Tillman, Nikolaus R McFarland, SH Subramony, Michael S Okun, Chris J Hass

**Affiliations:** Department of Kinesiology, Iowa State University, 235 Forker, Ames, IA 50011 USA; Department of Applied Physiology and Kinesiology, University of Florida, Gainesville, USA; Center for Movement Disorders and Neurorestoration, Department of Neurology, University of Florida, McKnight Brain Institute, Gainesville, USA; Department of Kinesiology and Health Promotion, Troy University, Troy, USA

**Keywords:** Finger tapping, Movement disorders, Movement rate, Movement amplitude, Coefficient of variation

## Abstract

**Background:**

Differentiating movement disorders is critical for appropriate treatment, prognosis, and for clinical trials. In clinical trials this is especially important as effects can be diluted by inclusion of inappropriately diagnosed participants. In early disease duration phases, disorders often have overlapping clinical features, such as impairments in repetitive finger movement, making diagnosis challenging. The purpose of this pilot study was to examine and compare repetitive finger movement performance in participants diagnosed with idiopathic Parkinson’s disease, Progressive Supranuclear Palsy, and spinocerebellar ataxias.

**Methods:**

Participants completed an unconstrained index finger flexion/extension movement (i.e. finger tap) in time with an incremental acoustic tone. Measures of movement rate, movement amplitude, and coefficient of variation were compared among groups.

**Results:**

Significant differences between groups were revealed for movement rate at faster tone rates. Participants with Parkinson’s disease tended to tap faster than the tone rate while participants with Progressive Supranuclear Palsy and spinocerebellar ataxia tended to tap slower. No significant differences were revealed for movement amplitude, but participants with spinocerebellar ataxia demonstrated greater variance in amplitude than participants with Parkinson’s disease.

**Conclusion:**

Quantitative analysis of repetitive finger movement performance at faster rates may be helpful to differentiate Parkinson’s Disease, Progressive Supranuclear Palsy and spinocerebellar ataxia.

## Background

While Parkinson’s disease (PD) is among one of the most common movement disorders, many other neurodegenerative syndromes display parkinsonian features and can present with similar deficits making accurate diagnosis challenging. Differentiation of other movement disorders from idiopathic PD is critical not only for appropriate treatment and prognosis, but also for clinical trials in which effects can be diluted by inclusion of inappropriately diagnosed subjects. As impaired control of repetitive finger movements can significantly impact the performance of daily living activities, such as writing and buttoning clothing in these patients, the performance of repetitive finger movements is a clinical tool used to assess severity, progression, and treatment efficacy of various movement disorders. Thus, the purpose of this study was to examine and compare repetitive finger movement performance among three different movement disorders that present with similar deficits in repetitive finger movement performance: idiopathic PD, Progressive Supranuclear Palsy (PSP), and spinocerebellar ataxias (SCA).

While persons with PD, PSP, and SCA may present with similar clinical deficits in repetitive finger movement performance, the etiology of these movement disorders differs. In general, PD is attributed to the loss of neurons in the basal ganglia [1], while SCA is attributed to the loss of neurons in the cerebellum [2]. PSP is attributed to a loss of neurons in the basal ganglia, brain stem, and cerebellum [3]. However, many other brain areas are impacted by these diseases, and changes in movement performance cannot not be solely attributed to a loss of neurons in one region of the brain [2-4]. In any case, the differing etiology may contribute to differences in repetitive finger movement performance when quantitatively evaluated.

Previous research has revealed that persons with PD demonstrate impairments in repetitive finger movements at tone rates near to and above 2 Hertz (Hz) (i.e. 2 beats per second) compared to healthy control participants [5-8]. Impairments in repetitive finger movement performance at tone rates near to and above 2 Hz were not improved with dopaminergic medication [5]. However, differential effects of deep brain stimulation of the subthalamic nucleus (STN-DBS) on movement rate and movement amplitude were revealed [7]. Specifically, STN-DBS improved movement amplitude but not movement rate at tone rates near to and above 2 Hz. This would suggest that other non-dopaminergic mechanisms may mediate the impairments of repetitive finger movements that emerge at tone rates near to and above 2 Hz and may attribute to the clinical similarity of repetitive finger movements in persons with PD, PSP, and SCA.

Research is limited regarding repetitive finger movement performance in persons with PSP and SCA. One study has examined differences in repetitive finger movement performance between participants with PD and PSP [9]. Ling and colleagues [9] revealed that the performance of repetitive finger movements as big and fast as possible can distinguish between these two movement disorders. It is unclear if there are differences in repetitive finger movement performance over a range of movement rates between participants with PD and PSP. Many persons with SCA also present with or include parkinsonian features, which can be confused with PD [10]. Yet, research examining differences in repetitive finger movement performance between participants with PD and SCA is limited. Only one study has compared repetitive finger movement performance at rates above and below 2 Hz in persons with PD and spinocerebellar degeneration [11]. Kosaka and collegues [11] revealed that performance differed between these two groups, suggesting that the cerebellum may play a role in the differential response. Research examining differences in repetitive finger movement performance in persons with PSP and SCA is limited.

Using the same paradigm in which the tone rate is systematically presented from low rates (i.e. 1 Hz) to high rates (i.e. 3 Hz), this study examined the differences in repetitive finger movement performance between participants with PD, PSP, and SCA across multiple tone rates. Given that 1) persons with PD, PSP, and SCA demonstrate similar clinical deficits in repetitive finger movement performance and 2) impairments in repetitive finger movements that emerge at rates near to and above in persons with PD do not improve with dopaminergic medication suggesting the involvement of non-dopaminergic mechanisms, we hypothesized that participants with PSP and SCA would also demonstrate changes in repetitive finger movement performance at tone rates near to and above 2 Hz. However, we also hypothesize that given the differing pathophysiological mechanisms of each movement disorder, the changes in repetitive finger movement performance at tone rates near to and above 2 Hz would differ among participants with PD, PSP, and SCA.

## Methods

### Participants

Nineteen participants with PD (16 male/3 female; mean age = 72 ± 9 years; 18 right handed/1 left handed; disease duration = 6 ± 4 years), 10 participants with PSP (3 male/7 female; mean age = 70 ± 4 years; 10 right handed; disease duration = 5 ± 4 years), and 12 participants with SCA (5 male/7 female; mean age = 50 ± 17 years; 10 right handed/2 left handed; disease duration = 7 ± 7 years) were recruited from the University of Florida Center for Movement Disorders and Neurorestoration in Gainesville, Florida. The UPDRS motor scores for the PD and PSP group tested off medication were 29 ± 12 and 43 ± 11 respectively. For the SCA group, two patients were diagnosed with SCA1, two with SCA2, three with SCA3, one with SCA6, one with SCA8, one with sporadic ataxia (onset after 50 years of age), and one with ataxia not otherwise specified. Diagnosis was confirmed by a fellowship-trained movement disorders neurologist. All participants were tested on medication as previous research demonstrated that the impairments in movement performance using the same task as in this study were not significantly affected by optimal medication [5]. No dyskinesias were noted during data collection. Participants with PD were tested on the most affected side (13 right side/6 left side) and participants with SCA and PSP were tested on the dominant side. All participants gave their written informed consent prior to inclusion into the study, and the University of Florida Institutional Review Board approved the procedures.

### Data collection

**Figure 1:**
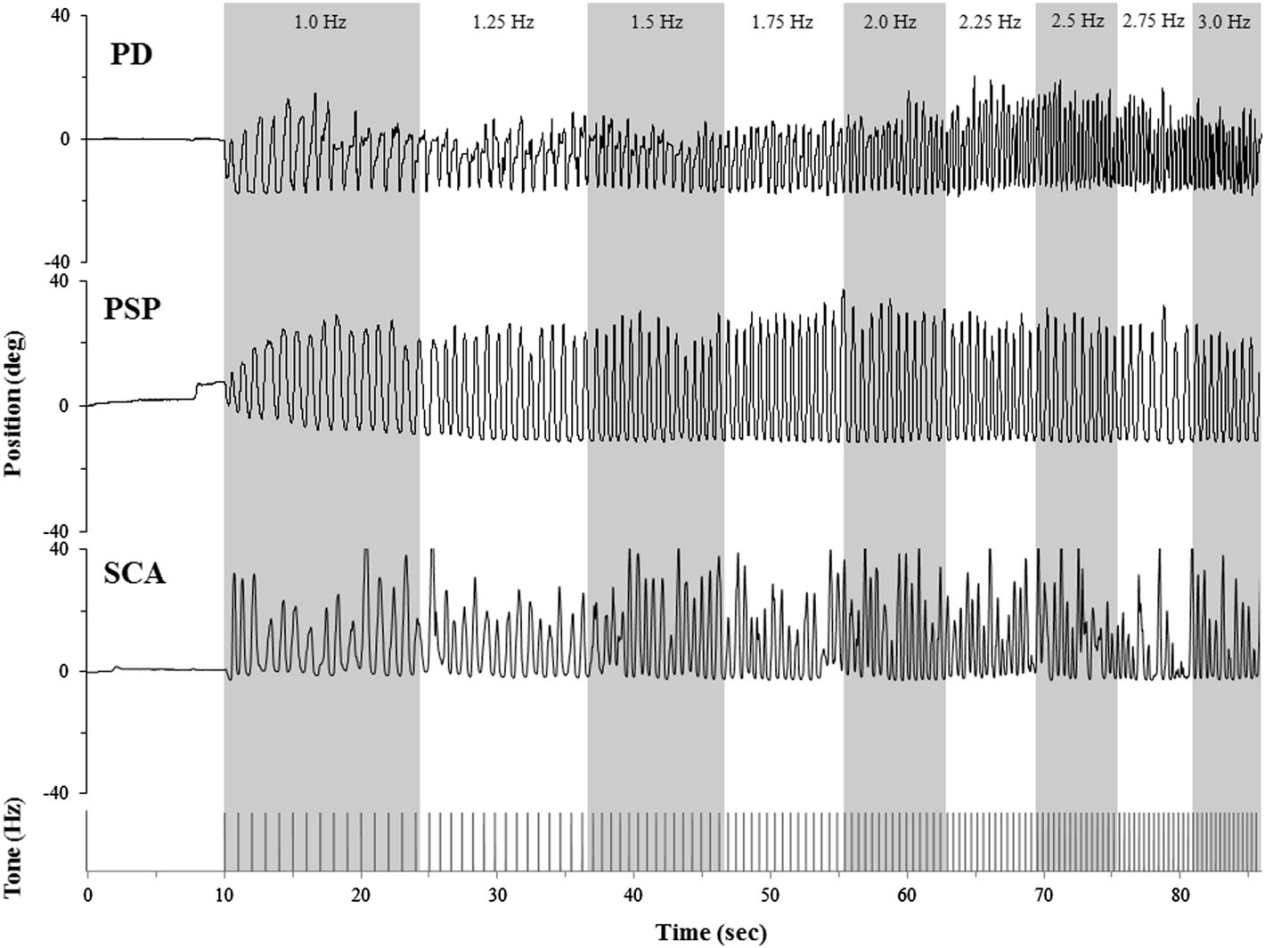
**Paradigm and raw data.** Paradigm example and position data from one participant with PD, one participants with PSP, and one participant with SCA.

All participants completed three trials of an unconstrained finger flexion-extension movement (i.e. finger tap) in time with a series of acoustic tones presented at 1 Hz for 15 intervals and then increased by 0.25 Hz every 15 intervals until reaching 3 Hz [5-7] (Figure 1). The forearm (placed in a pronated position), wrist, thumb, and fingers 2–4 were supported by a brace. The index finger remained unconstrained for full range of motion. A goniometer was used to collect position of the index finger. Movement rate, peak-to-peak amplitude, and corresponding coefficient of variation (CV) were obtained for each movement and averaged across each tone rate. Movement amplitude was normalized to the amplitude at 1 Hz to allow for comparison across tone rates since no constraints were placed upon range of motion. Movement rate difference (MRΔ) was calculated as the difference between the given tone rate and actual movement rate.

### Statistical analyses

To examine repetitive finger movement performance in the PSP and SCA groups alone, a single factor repeated measures analysis of variation (ANOVA) model was completed to estimate differences in MRΔ and normalized peak-to-peak amplitude across tones rates for each group. To examine differences between MRΔ, normalized peak-to-peak amplitude and CV across all groups, a repeated measures ANOVA model was estimated. The between-group factor was PD vs. PSP vs. SCA, and the within-subjects factor was tone rate. Post hoc analysis was completed using Tukey’s Honestly Significant Difference test. The level of significance was set at α < 0.05.

## Results

Figure 1 shows raw position data from one participant with PD, one participant with PSP, and one participant with SCA. Note that at approximately 2.25 Hz and above the participant with PD demonstrates an increase in movement rate with little decrement in movement amplitude. In contrast, the participant with PSP does not increment movement rate starting at approximately 2.0 Hz and maintains a relatively stable movement rate and movement amplitude until reaching 3.0 Hz. Finally, the participant with SCA demonstrated a much greater variation in movement performance across all tone rates, and at rates near to and above 2.0 Hz, this participant remains slower than the intended tone.

**Figure 2:**
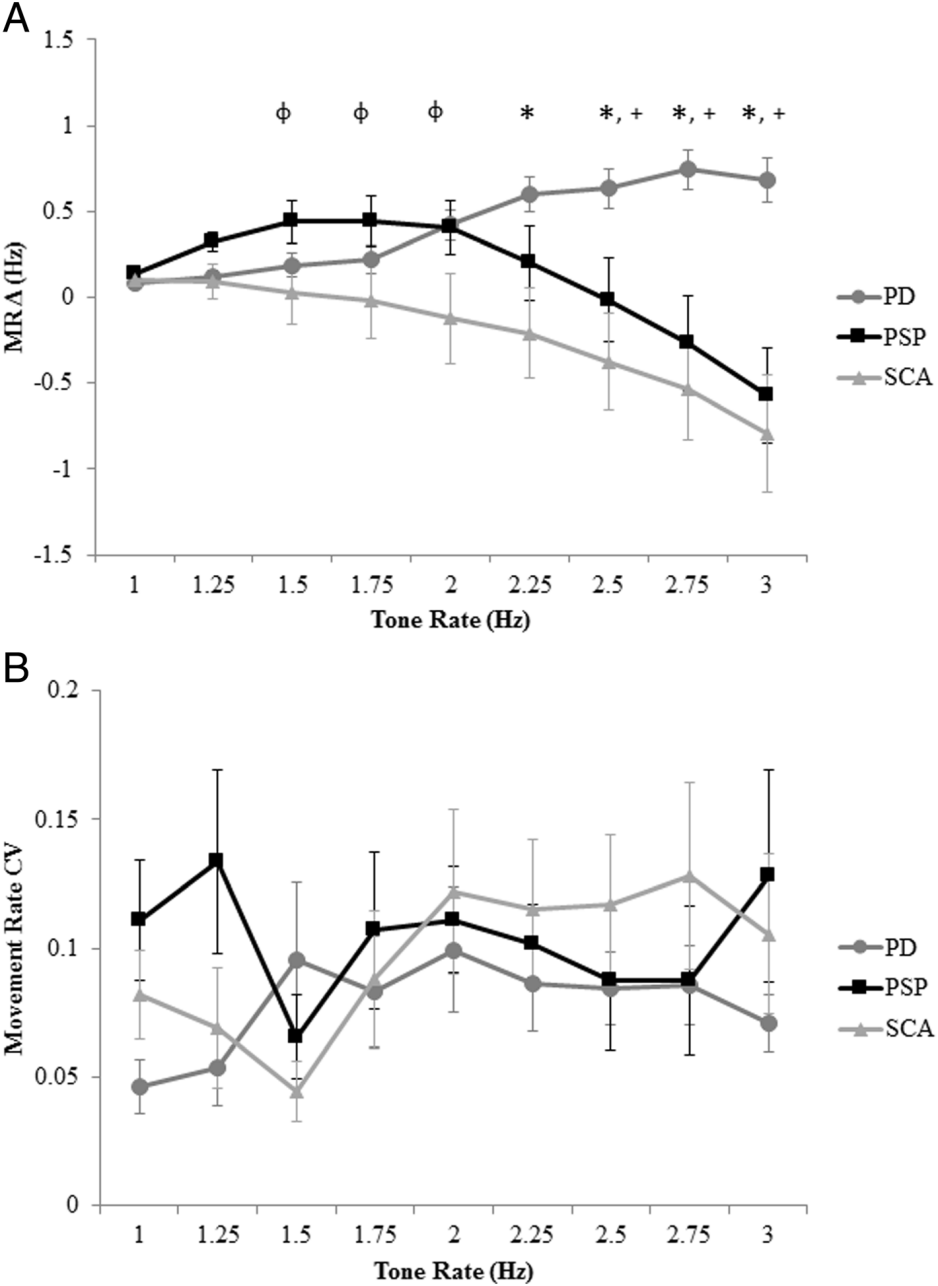
**Movement rate difference and movement rate coefficient of variation.** Mean and standard error for **(A)** movement rate difference (MRΔ) and **(B)** movement rate coefficient of variation across all tone rates for the PD, PSP, and SCA groups. Crossed (^+^) designate significant differences between the PD and PSP groups. Asterisks (*) designate significant differences between the PD and SCA groups. Phi (^φ^) designates significant differences between the PSP and SCA groups.

For comparison of the PSP and SCA groups alone, there was a significant effect of MRΔ for the PSP group (F(8) = 7.48, p < 0.001) and the SCA group (F(8) = 13.809, p < 0.001) (Figure 2A). For the PSP group, post hoc analysis revealed that MRΔ significantly differed between tone rates of 1.5 Hz and 3.0 Hz (p = 0.03), and 1.75 Hz and 3.0 Hz (p = 0.05) only. For the SCA group, post hoc analysis revealed that MRΔ significantly differed 1) from 1 Hz at tone rates of 2.75 Hz and above (p < 0.01), 2) from 1.25 Hz at tone rates of 2.75 Hz and above (p < 0.01), 3) from 1.5 Hz at tone rate of 2.75 Hz and above (p < 0.03), 4) between 1.75 Hz and 3.0 Hz (p < 0.001), 5) between 2.0 Hz and 3.0 Hz (p = 0.004), and between 2.25 Hz and 3.0 Hz (p = 0.02). There were no significant differences in normalized peak-to-peak amplitude for either the PSP (F(8) = 0.02, p = 0.89) or SCA (F(8) = 0.17, p = 0.69) group.

When comparing performance across groups, it was observed that MRΔ between the three groups diverged at tone rates near to 2 Hz. Specifically, the PD group moved faster than the intended tone rate while both the PSP and SCA group moved slower than the intended tone rate (Figure 2A). Moreover, for both MRΔ and normalized peak-to-peak amplitude the standard error was larger in the PSP and SCA groups than the PD group. Thus, a comparison of CV for both outcome measures was completed.

Statistical comparison revealed a significant rate effect (F(8) = 7.12, p < 0.001), a significant group effect (F(2) = 8.21, p = 0.001), and a significant interaction effect (F(16) = 13.28, p < 0.001) for MRΔ. Post hoc analysis revealed that MRΔ between the PD group significantly differed (moved faster) from the PSP group at tone rates of 2.5 Hz and above (p < 0.02) and significantly differed (moved faster) from the SCA group at tone rates of 2.0 Hz and above (p < 0.02). MRΔ between the PSP group significantly differed from the SCA group at tone rates of 1.5 Hz, 1.75 Hz, and 2.0 Hz (p < 0.05) (Figure 2A). For CV of MRΔ, there was no main effect of tone rate, group, or interaction effect (Figure 2B).

**Figure 3:**
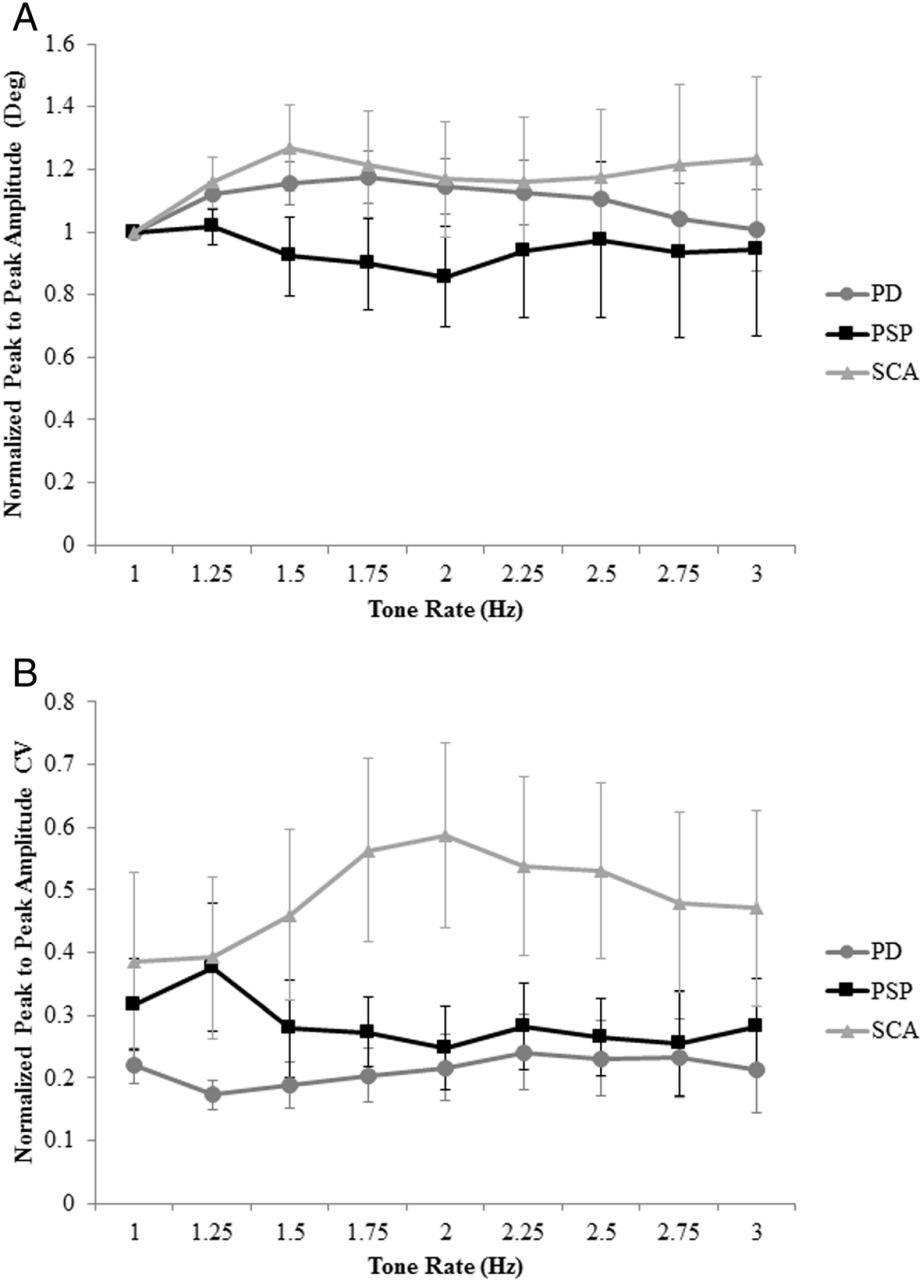
**Amplitude and amplitude coefficient of variation.** Mean and standard error for **(A)** normalized peak-to-peak amplitude and **(B)** normalized peak-to-peak amplitude coefficient of variation (CV) across all tone rates for the PD, PSP, and SCA groups.

Figure 3 shows the normalized peak-to-peak amplitude and CV for all three groups. No main effect of tone rate or group, as well as no interaction effect, was revealed for normalized peak-to-peak amplitude (Figure 3A). However, there was a main effect of group for CV of normalized peak-to-peak amplitude. Amplitude variance in the SCA group significantly differed from the PD group (p = 0.04). There were no differences in amplitude variance between the SCA and PSP groups (p = 0.26) or the PSP and PD groups (p = 0.81). There was no main effect of tone rate or interaction effect for normalized peak-to-peak amplitude CV (Figure 3B).

## Discussion

This study is the first to examine differences in repetitive finger movement performance in persons with PD, PSP, and SCA. Results revealed significant differences in movement rate across groups and tone rates. The PD group tended to move faster than the tone, while the PSP and SCA groups moved slower than the intended tone at higher tone rates. While there were no differences among groups or across tone rates in movement amplitude, the SCA group demonstrated greater variance in movement amplitude compared to the PD group. Taking these characteristics together, the results of this study suggest that quantitatively evaluating the movement rate and amplitude variance of repetitive finger movement performance at high rates may help distinguish among these three movement disorders. Future work should focus on testing patients early in disease and off medication.

There were no differences observed in movement amplitude across tone rates or across groups in this study. All participants were tested on medication which may account for the lack of detectable effect. However, when comparing peak-to-peak amplitude (not normalized to 1 Hz) across groups, peak-to-peak amplitude was largest across all tone rates in the SCA group (27.0 ± 0.6 degrees) and smallest in the PSP group (16.2 ± 0.8 degrees) compared to the PD group (21.3 ± 0.5 degrees). This is in keeping with previous research that has reported decreased movement amplitude for repetitive finger movements in persons with PSP compared to persons with PD [9]. No study has compared movement amplitude of repetitive finger movements between PD and SCA. However, the increased variance in movement amplitude for the SCA group compared to the PD group is consistent with previous findings comparing gait in PD and cerebellar ataxia [12], and may reflect ataxia/poor coordination. The main differences in repetitive finger movement performances between the PD, PSP, and SCA groups were related to movement rate.

**Figure 4:**
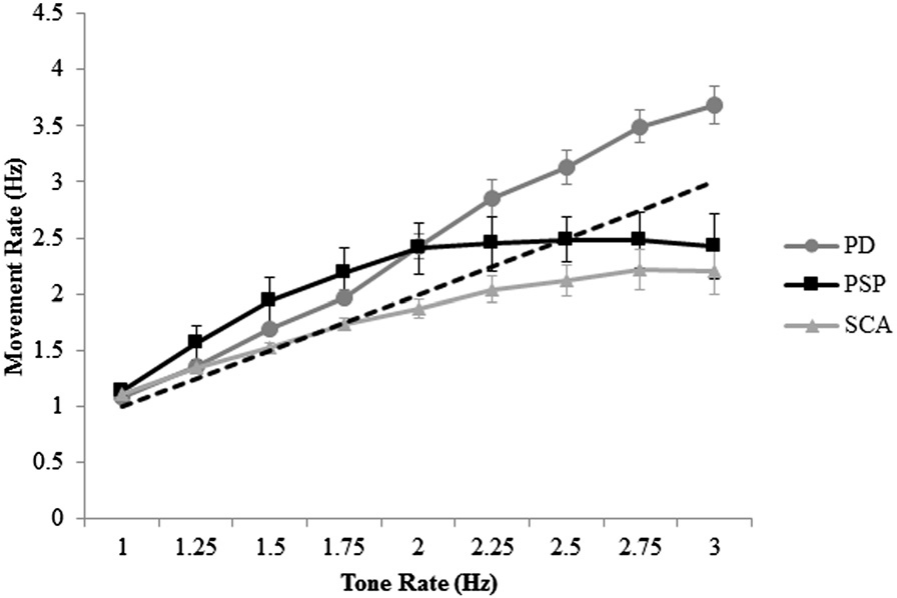
**Movement rate.** Mean and standard error for movement rate across all tone rates for PD, PSP, and SCA groups.

A major finding of this study is that differences in movement rate among groups emerged at tone rates above 2 Hz. Results revealed that the PSP and SCA groups moved slower than the intended tone while the PD group moved faster than the intended tone at higher tone rates. However, the negative MRΔ for the PSP and SCA groups may be due to an inability to sustain movement rates above a certain frequency due to incoordination or rigid-bradykinesia. Indeed, participants with PSP incremented movement rate with the tone rate until reaching the tone rate of 2 Hz. At rates above 2 Hz, the PSP group maintained a constant movement rate (2.4 to 2.5 Hz with a standard error of 0.22 to 0.29). In fact, the PSP group did not fall below the intended rate until reaching the tone rate of 2.5 Hz (see Figure 4). Thus, the PSP group demonstrated an inability to increment movement rate above 2.5 Hz. A previous study examining repetitive finger movements in persons with PSP describes the finger tap pattern as “hypokinetic without decrement” [9]. Ling and colleagues [9] further suggest that persons with PSP demonstrate a lack of decrement in movement amplitude during repetitive finger movement compared to persons with PD. Likewise, in the present study, there was no significant change in movement amplitude across tone rates in the PSP group. Taken together, persons with PSP may lock into a steady state performance of repetitive movement, potentially perseverating more, that is unaffected by changing environmental cues (increase in tone rate).

In contrast to the PSP group, the SCA group remained slower than the intended tone rate beginning at 2 Hz, but were still able to increment movement rate minimally (1.8 to 2.2 Hz with a standard of 0.06 to 0.22) (see Figure 4). This is in keeping with previous studies. Akhlaghi and collegues [13] demonstrated that persons with Friedreich’s ataxia demonstrated a slower finger tapping rate when compared to healthy control participants [13]. Kosaka and collegues [11] have demonstrated that at rates above 2 Hz, persons with spinocerebellar degeneration demonstrated a decreased movement rate [11]. Interestingly, gait speed has been shown to affect gait variability in persons with cerebellar ataxia, with the highest variability occurring during slow and fast walking [14,15]. In the current study, no significant change in movement amplitude across tone rates was observed, but amplitude variance was significantly increased compared to the PD group. The variance in both movement rate and amplitude in the SCA group did increase, though not significantly, as the tone rate increased. Thus, in this study, we suggest the reduced movement rate in the SCA group may be the result of participants trying to reduce movement variability to allow for a more stable overall movement performance.

Previous research has reported that persons with PD demonstrate impairments in repetitive finger movement performance, characterized by a dramatic increase in movement rate accompanied by hesitations and arrest as well as a loss of movement amplitude, at tone rates near to and above 2 Hz and is not improved with dopaminergic medication [5-8]. This would suggest that non-dopaminergic mechanisms may contribute at least in part to this impairment [5]. In healthy populations, transition in repetitive finger movement performance at rates near to and above 2 Hz is associated with changes in activity over primary and secondary motor areas, as well as changes in motor cortical oscillations [16-18]. This may suggest that a change in motor control of repetitive finger movements, potentially from discrete to continuous movement, occurs at rates near to 2 Hz. The change in motor control may lead to the deterioration of repetitive finger movement performance in persons with PD, as well as, PSP and SCA. In this study, movement rate performance in the PSP and SCA groups diverged from the PD group near 2 Hz. These results indeed support the hypothesis that control of repetitive finger movement performance changes at rates near 2 Hz and may be mediated in part by cerebellar function as cerebellar dysfunction has been previously reported in PD, PSP, and SCA [2-4]. However, given the differences in movement performance across the groups, pathophysiological mechanisms involving cerebellar and basal ganglia function may contribute differentially to the changes in movement performance at high movement rates.

Our study had several limitations. First, as this was a pilot study the sample size was small for each of the three populations studied. Due to the small sample size we were unable to match subjects for age, gender, or disease severity (further difficult given the inherent differences among these disorders), and the individual participant variability may have impacted statistical significance. Second, the SCA group in general was more heterogeneous than the PD or PSP groups and may account in part for the increased variation seen in this group. Third, subjects were evaluated in mid to late disease (average disease duration was at least 5 years for each group). Examining these patients earlier in disease, during the first or second year post-diagnosis, may be more clinically meaningful and could support this approach as a potential diagnostic tool if these findings are validated. There is a possibility that fatigue may have contributed to the decrements in movement performance. Previous research has shown that impairments in repetitive finger movement performance in persons with PD using this same task was not due to fatigue [6]. However, the contribution of fatigue to impairments in persons with PSP or SCA has not been tested. Finally, cognitive impairment is often present in patients with PD, PSP, and SCA, and could potentially affect the variability in the results. No cognitive information was collected in this study to rule this potential confound out. However, the task in this study was rather simple and involved limited cognitive effort.

## Conclusion

The results of this study suggest that persons with PD, PSP, and SCA may use different motor control strategies for repetitive finger movement performance at high movement rates. Specifically at movement rates near 2 Hz and above, participants with PD tended to move faster than the intended tone (similar to festination); participants with PSP maintained a constant rate; and participants with SCA moved slower than the intended tone though still minimally incremented movement rate and demonstrated an increase in variance in movement amplitude. Future imaging studies are needed to examine if these differences in movement performance are accounted for by differing pathophysiological mechanisms. However, this study provides initial evidence that quantitatively evaluating repetitive finger movement performance has the potential to differentiate movement disorders that may present similar to PD.

## Abbreviations

PD: Parkinson’s disease
PSP: Progressive Supranuclear Palsy
SCA: Spinocerebellar ataxia
HZ: Hertz
STN-DBS: Deep brain stimulation of the subthalamic nucleus
CV: Coefficient of variation
MRΔ: Movement rate difference
ANOVA: Analysis of variation
